# Allometry of individual reproduction and defense in eusocial colonies: A comparative approach to trade-offs in social sponge-dwelling *Synalpheus* shrimps

**DOI:** 10.1371/journal.pone.0193305

**Published:** 2018-03-14

**Authors:** Sarah L. Bornbusch, Jonathan S. Lefcheck, J. Emmett Duffy

**Affiliations:** 1 Department of Evolutionary Anthropology, Duke University, Durham, NC, United States of America; 2 Department of Biological Sciences, Virginia Institute of Marine Science, The College of William & Mary, Gloucester Point, VA, United States of America; 3 Bigelow Laboratory for Ocean Sciences, East Boothbay, ME, United States of America; 4 Tennenbaum Marine Observatories Network, Edgewater, MD, United States of America; University of Pretoria, SOUTH AFRICA

## Abstract

Eusociality, one of the most complex forms of social organization, is thought to have evolved in several animal clades in response to competition for resources and reproductive opportunities. Several species of snapping shrimp in the genus *Synalpheus*, the only marine organisms known to exhibit eusociality, form colonies characterized by high reproductive skew, and aggressive territoriality coupled with cooperative defense. In eusocial *Synalpheus* colonies, individual reproduction is limited to female ‘queens’, whose fecundity dictates colony growth. Given that individual reproduction and defense are both energetically costly, individual and colony fitness likely depend on the optimal allocation of resources by these reproducing individuals towards these potentially competing demands. *Synalpheus* species, however, display varying degrees of eusociality, suggesting that reproducing females have adopted different strategies for allocation among reproduction and defense. Here, we use structural equation modeling to characterize the relationships between the allometry of queen reproductive capacity and defensive weaponry, and colony size in six eusocial *Synalpheus* species, estimating trade-offs between reproduction and defense. We document strong trade-offs between mass of the fighting claw (defense) and egg number (reproduction) in queens from weakly eusocial species, while the trade-off is reduced or absent in those from strongly eusocial species. These results suggest that in less cooperative species, intra-colony conflict selects for queen retention of weapons that have significant costs to fecundity, while reproducing females from highly eusocial species, i.e., those with a single queen, have been able to reduce the cost of weapons as a result of protection by other colony members.

## Introduction

The shift from a solitary life to group living represents one of a handful of key transitions in the evolution of life [1]. Eusociality, one of the most extreme forms of social organization, is traditionally recognized in groups of organisms that display three key characteristics: overlap of generations, cooperative brood-care, and non-reproducing or sterile worker castes [2,3] Accordingly, most members in eusocial colonies are physiologically or behaviorally sterile, forgoing individual reproduction in order to raise the offspring of others and cooperatively defend the colony. Kin selection, which favors behavior that promotes the reproductive success of close relatives, offers a well-supported explanation for the evolution of sterility in altruistic social organizations [4,5]. Many communities of eusocial species, however, vary in the proportion of reproductively-active individuals, suggesting that despite strong kin selection, competition for reproductive resources continues to shape social structure to some degree.

Eusociality has evolved multiple times, including in several lineages of Hymenoptera, Isoptera, and other insect groups [6], two species of mole rats [7], and at least four lineages within the marine shrimp genus *Synalpheus* [8–10]. Certain characteristics of sponge-dwelling *Synalpheus* make the genus a useful model for examining the evolution of social behavior. These characteristics include a broad range of social organization, relatively few species within the sponge-dwelling clade (~40), and similarities in general morphology and ecology, such as a sedentary and symbiotic lifestyle [9,11]. *Synalpheus* shrimps reside within the canals of host sponge species in what appears to be a parasitic or possibly mutualistic relationship [12,13]. This sedentary lifestyle promotes localized dense populations as well as strong competition for space. The limited availability of sponges [14] and generally aggressive territoriality of sponge-dwelling shrimp [15] likely inhibits the success of dispersing individuals at forming new colonies, reinforcing the advantages of kin aggregation [16,17].

The reef matrix, including *Synalpheus* host sponges, is densely populated with a wide variety of macroinvertebrates competing for space and resources. Most of the sponge species used as hosts by these shrimps are occupied, such that few are available for colonization and *Synalpheus* experience regular conflict in obtaining and defending sponge habitats. Competition for these scarce resources has likely selected for cooperative defense in multiple eusocial *Synalpheus* species [14,15], in which the large fighting claw (major chela) figures prominently. This hypothesis is consistent with high levels of cooperative defense and highly aggressive inter- and intraspecific conflict [15,18–20]. *Synalpheus* colony members aggressively defend the sponge boundaries from intruding organisms, including non-colony conspecifics.

Despite experiencing generally similar lifestyles and ecological pressures, the degree of eusociality, expressed as reproductive skew (the ratio of reproducing females, or queens, to non-reproducing colony members), varies across cooperative *Synalpheus* species. This variation in the density of reproducing females raises interesting questions about the role of intra-sexual competition in shaping eusocial evolution. Namely, how does the presence of a single or multiple queens influence their reproductive and defense capabilities, and, in turn, the overall colony growth? Here we consider individual ‘reproduction’ to be a queen’s fecundity and contribution to colony growth. In colonies of *S*. *elizabethae*, the presence of a queen inhibits the reproductive maturation of otherwise reproductively capable females, even in the absence of queen aggression or policing [21]. This suggests a facultative, and perhaps chemical, mechanism of reproductive monopolization. In contrast, research on other *Synalpheus* species demonstrates a positive relationship between reproductive skew and defensive weaponry, suggesting that queen defensive allometry is linked to direct female-female competition over individual reproduction [22]. While this and other evidence indicates that intraspecific competition for limited resources plays a crucial role in the evolution of eusociality and caste formation in certain eusocial insects [23], the role of intrasexual competition among colony members in shaping individual and colony level characteristics in eusocial *Synalpheus* species remains unclear. Exploring this question is of great interest since *Synalpheus* shrimps are closer to the demographic and ecological conditions at the origin of eusociality than are the better known, but more ancient, lineages of eusocial insects.

Given that eusocial colonies often benefit from larger colonies for cooperative defense, foraging, and habitat maintenance, the reproducing female(s) are expected to face strong selection for fecundity and therefore to incur higher energy requirements compared with non-reproductives. In addition to being responsible for all colony growth, queens in social shrimp species often possess equally large defensive weaponry as non-reproducing colony members (with the exception of *S*. *filidigitus* queens who do not possess a major chela) [11], suggesting that queens also engage in aggressive behavior and defense. Queen involvement in colony defense is likely high during colony founding and in smaller colonies generally, and decreases as colony size increases. Given finite resources and energy, and that both reproduction [24–26] and defense [27–29] are considered energetically costly to an individual, investment in these behaviors likely entails an energy allocation trade-off for *Synalpheus* eusocial queens. Moreover, optimal resolution of that trade-off is likely to differ in species with different degrees of cooperation and colony size. Here we suggest that this trade-off can be recognized as a negative correlation between queen chela mass (defense) and egg number (reproduction), and we explore how this trade-off varies among species at different levels of social development.

Although eusocial *Synalpheus* queens are often the largest individuals in a colony [30,22], because individuals cooperate to varying degrees within social shrimp colonies, we can expect that a queen’s allometry and reproductive success will vary depending on the size of the colony and her interactions with other members. In eusocial colonies with multiple reproducing females, reproduction is shared among queens, who contribute to the colony’s growth. In these species, reproduction by multiple queens may provide an ecological advantage at the colony level by increasing the number of individuals that can defend the colony and releasing the queens from the pressures of defense [31–33]. However, as multi-female colonies grow, the presence of multiple queens may increase female-female conflict to maintain reproductive status, causing queens to invest more in defensive weaponry even if inter-colony conflict decreases. Thus, we would expect individual queen allometry to be linked to both colony size and reproductive skew. Here, we explore these ideas by comparing the traits of queens from *Synalpheus* species with multiple reproducing females, considered weakly eusocial, to those from species with a single or few queens, which are considered strongly eusocial [34].

Given the high energetic cost of both reproduction and defense, a queen’s allocation of energy between intra-sexual competition for reproductive resources (defense) and individual fecundity (reproduction) is expected to be influenced by the degree of eusociality of each *Synalpheus* species. Specifically, in large eusocial colonies with a single queen and many “workers”, queens are expected to have neither a need to defend against intruders, which is accomplished by other colony members, nor to compete for reproductive monopoly, both of which relax selection to maintain a large fighting claw. Under this scenario, we would expect a diminished trade-off between queen reproduction and defense, and a decline in claw size in favor of allocation to fecundity. In colonies that are smaller and/or have multiple queens, selection is expected to maintain the queen’s fighting claw for purposes of defense against intruders and/or competition for reproduction. This maintenance of defensive weaponry is predicted to decrease potential investment in fecundity, reinforcing the trade-off between reproduction and defense in weakly eusocial species. We explored these predictions by studying reproducing females from six species of *Synalpheus*, all considered eusocial but varying in the degree of reproductive monopoly within the colony. We quantified the relationships between queen reproduction and defense, and colony size in the six species using structural equation modeling to disentangle the direct and indirect controls on this trade-off.

## Methods

### Synalpheus collections

*Synalpheus* individuals used for this study were collected at multiple Caribbean sites over the course of 25 years between 1988 and 2013: Panama (1988, 1991–1993, 2007–2009, 2011), Belize (1993–1996, 1998, 1999, 2001–2005), Bahamas (2001), Jamaica (2008), and Florida (1995, 2005–2006, 2013). All collections were completed with the approval of the appropriate regulating organizations: Direccion General de Ordenacion y Manejo Intregral de la Autoridad de los Recursos Acuaticos de Panama (Panama), Department of Fisheries (Belize), The Bahamas Environment, Science & Technology (BEST) Commission, The Ministry of The Environment (Bahamas), National Environment & planning agency (Jamaica), and Florida Fish and Wildlife (Florida). The species and protocols used in this study did not require approval from the Institutional Animal Care and Use Committee.

Collection of sponges and coral rubble harboring shrimps was carried out on coral reefs via SCUBA or snorkeling [12]. Because *Synalpheus* colonies inhabit the internal canals of marine sponges, the sampling of complete colonies required the collection of the host sponges. In some cases, the sampling of whole sponges was limited in order to preserve reef integrity and minimize disturbance. In the laboratory, sponges were dissected and all individuals of all shrimp species were removed. Shrimp were identified to species and reproductive status was recorded (e.g. non-reproducing male or female, or ovigerous female). Specimens were preserved, usually in 95% ethanol.

### Study specimens

A total of 353 egg-bearing females from 221 colonies across the six eusocial species were analyzed: *Synalpheus brooksi* (n = 121), *S*. *chacei* (n = 59), *S*. *elizabethae* (n = 51), *S*. *microneptunus* (n = 21), *S*. *rathbunae* (n = 49), and *S*. *regalis* (n = 52). The specimens were chosen from the collection using the following criteria: the female must be (1) ovigerous and (2) have a major chela, and (3) the entire colony must have been collected with the host sponge. Reproducing females show no indication of seasonal breeding cycles: ovigerous females from each species were recorded throughout any given year. Because colonies were necessarily sampled destructively and at discrete time points, longitudinal data on female egg production was unavailable. By using multiple females from each species as replicates, we aimed to sample a representative window of queen development and egg production. Colony size varied with a maximum of 398 individuals and included up to 27 reproducing females. Because immigration and emigration appear rare in *Synalpheus* colonies, we expect colony size to be directly related to queen reproduction [8], i.e., we can infer that larger colonies are older. It has not been possible, however, to measure the age of colonies or their members directly.

Although members of eusocial *Synalpheus* species are generally smaller than those of pair-forming and non-eusocial species, eusocial queen body and chela size vary widely across species [30]. The most extreme form of the trade-off between chela size and reproduction is seen in the queens of *Synalpheus filidigitus*, which produce large clutches and possess two equally-sized small chelae [11]. The lack of major chela seen in *S*. *filidigitus* queens, however, makes them unsuitable for comparison to the other species and therefore this species excluded from this analysis. The six species included here are from three distinct clades within the gambarelloides group of the genus *Synalpheus* [35,36], and so represent three distinct origins of eusociality within the genus. To study the reproductive and colony characteristics of the six species, preserved ovigerous females were photographed and measured using light microscopy.

### Photographing and measuring specimens

**Fig 1:**
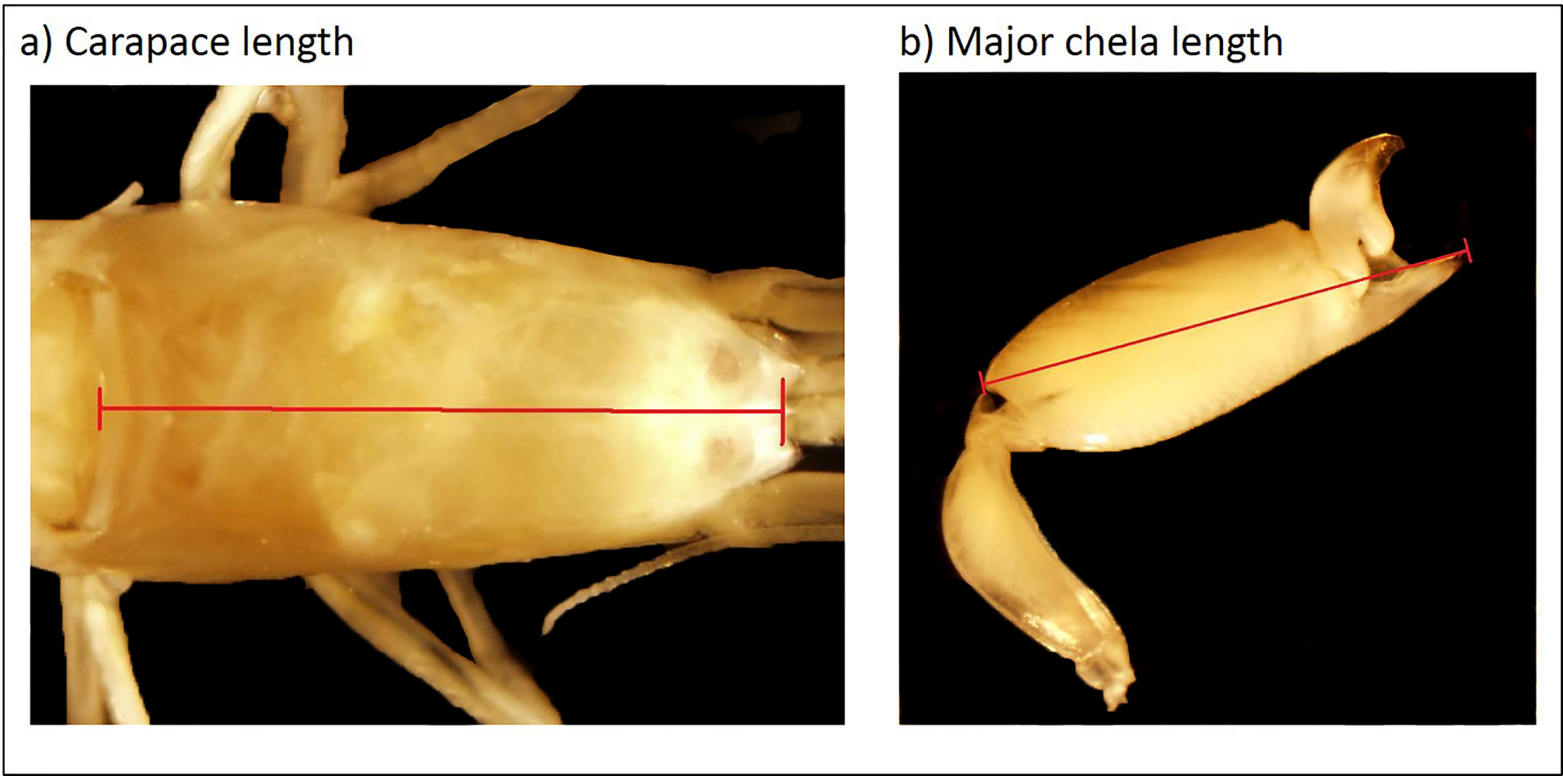
**Allometric measurements for (a) carapace length and (b) chela length on preserved, sponge-dwelling shrimp**. Measurements in millimeters were taken along the line shown using ImageJ.

Queen body and chela size were determined using photographic measurements performed in ImageJ 1.x [37]. Length of the carapace, commonly used as an index of body size among decapod crustaceans, was photographed dorsally with the rostrum visible and measured, to the nearest millimeter, from the center point between the valleys on either side of the rostrum to the center of the posterior edge of the carapace (Fig 1A). The major chela was removed from the specimen’s thorax, photographed, and measured from the tip of the fixed finger to the opposite dorsal end of the palm of the chela (Fig 1B).

We measured reproductive output of a queen as the number of embryos (hereafter, egg number) carried in her brood pouch. All eggs were removed from each female’s egg pouch, photographed, and counted.

### Individual allometry calculations

In order to quantify claw allometry and reproductive capacity, a combination of individual morphological variables and colony-level characteristics were calculated. Lengths of carapace and major chela were measured for every specimen. From these, major chela mass and body mass (excluding chela mass) were calculated using formulae in Duffy & Macdonald 2010 [14]:

$$Body\;mass=0.5968*e^{\left(0.4892*carapace\;length\right)}$$ (1)

$$Major\;chela\;mass=0.3135*e^{\left(0.4268*major\;chela\;length\right)}$$ (2)

### Colony level variables

We measured two variables at the colony level: colony size and an index of eusociality. Colony size is the total number of individuals of the same species found within a single sponge. The eusociality index (*E*) for each colony was calculated using the number of ovigerous females and colony size (formula derived from Chak et al 2017). Mean *E* for each of the six species was then calculated as the average *E* for all colonies of a given species [34,38]:

$$E=1-(\frac{2*number\;of\;queens}{colony\;size})$$ (3)

### Statistical analysis

**Fig 2:**
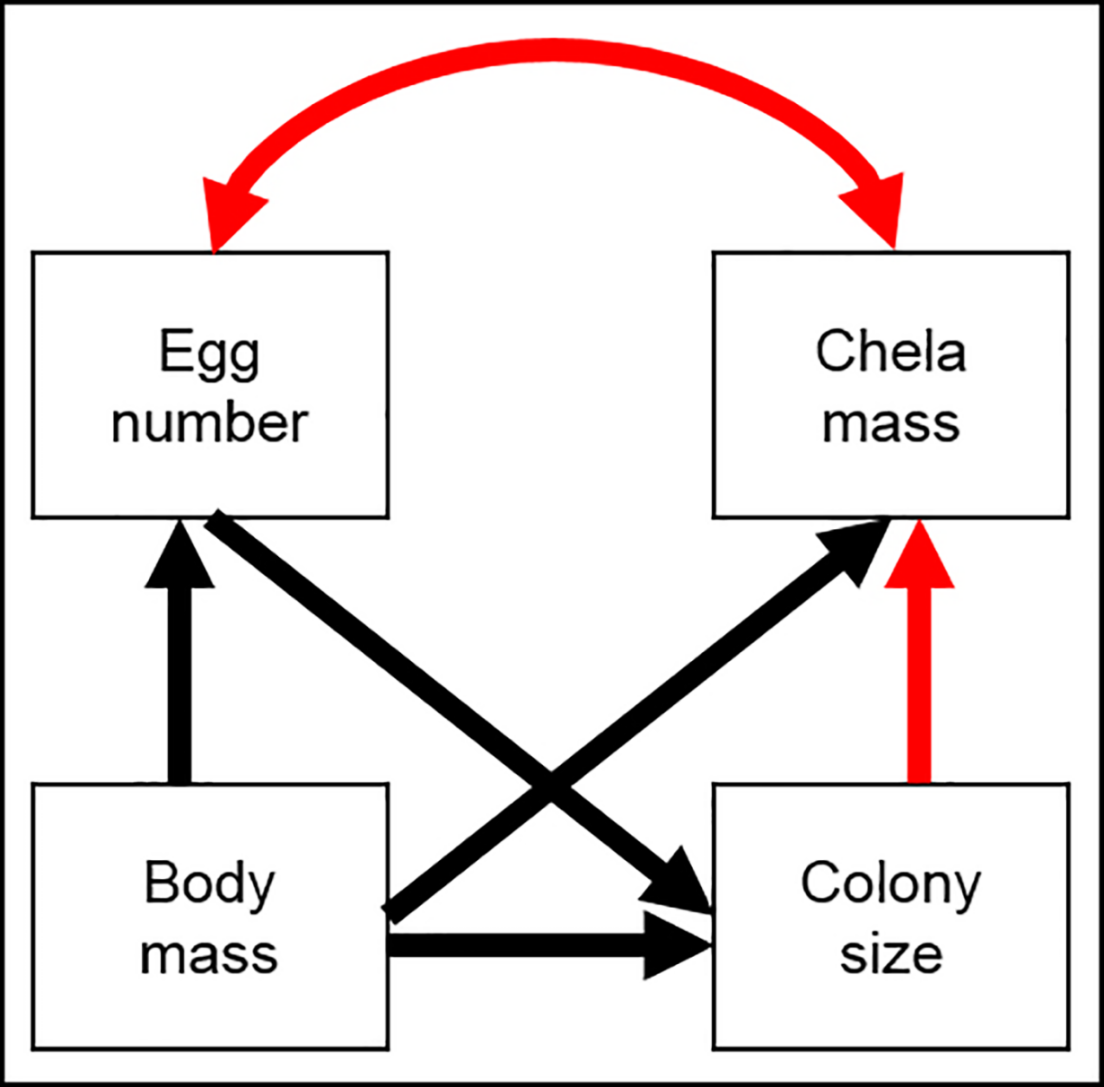
Conceptual path diagram showing the hypothesized directional relationships among variables. The sign of the proposed relationship is given and color coded: (positive/negative, +/-, black/red).

We modeled the relationships between species means of queen body mass, reproductive capacity (clutch size), queen defense capacity (chela size), and colony size. To approximate a normal distribution, all variables were first log_10_-transformed. We then characterized the relationships among all variables in a single causal network using structural equation modeling [39]. Structural equation modeling has several advantages over a univariate approach, principally that multiple hypotheses, such as those regarding allocation to reproduction versus defense, can be evaluated simultaneously. Similarly, it allows estimation of the bivariate trade-off among these two indicators, while accounting for the directional influence of other covariates in the model (Fig 2; double-headed arrow). The trade-off, estimated as a partial correlation, between reproduction (egg number) and defense (major chela size) is of principal interest, since it directly quantifies the degree to which reproduction is influenced by investment in defensive weaponry.

We employed the confirmatory mode of structural equation modeling, in which the direction of causality among variables was hypothesized *a priori* and then tested using data. Therefore, the same model configuration was fit for all six species in a single multigroup analysis [39]. Because we were specifically interested in estimating all possible relationships among the four variables in our analysis, we evaluated a fully saturated structural equation model (SEM). Consequently, typical goodness-of-fit tests, e.g., χ^2^ tests, cannot be performed. In lieu of such tests, we evaluated the significance and proportion of variance explained (R^2^) for individual paths. For each model, we report both the unstandardized and standardized effect sizes (path coefficients, S1 Table). Unstandardized coefficients are presented in the original units of the variable and therefore allow for comparison of the absolute strength of a given relationship (path) among the different species. Standardized coefficients, which standardize the units of all paths within one model, allow for comparison of the relative strengths of different pathways within one species. Experiment-wide threshold for Type I error was set at α = 0.05, and all SEMs were evaluated using the *lavaan* package R [40]. All data and R scripts to reproduce the analysis are given in the supplementary materials (Supplement 1).

## Results

**Fig 3:**
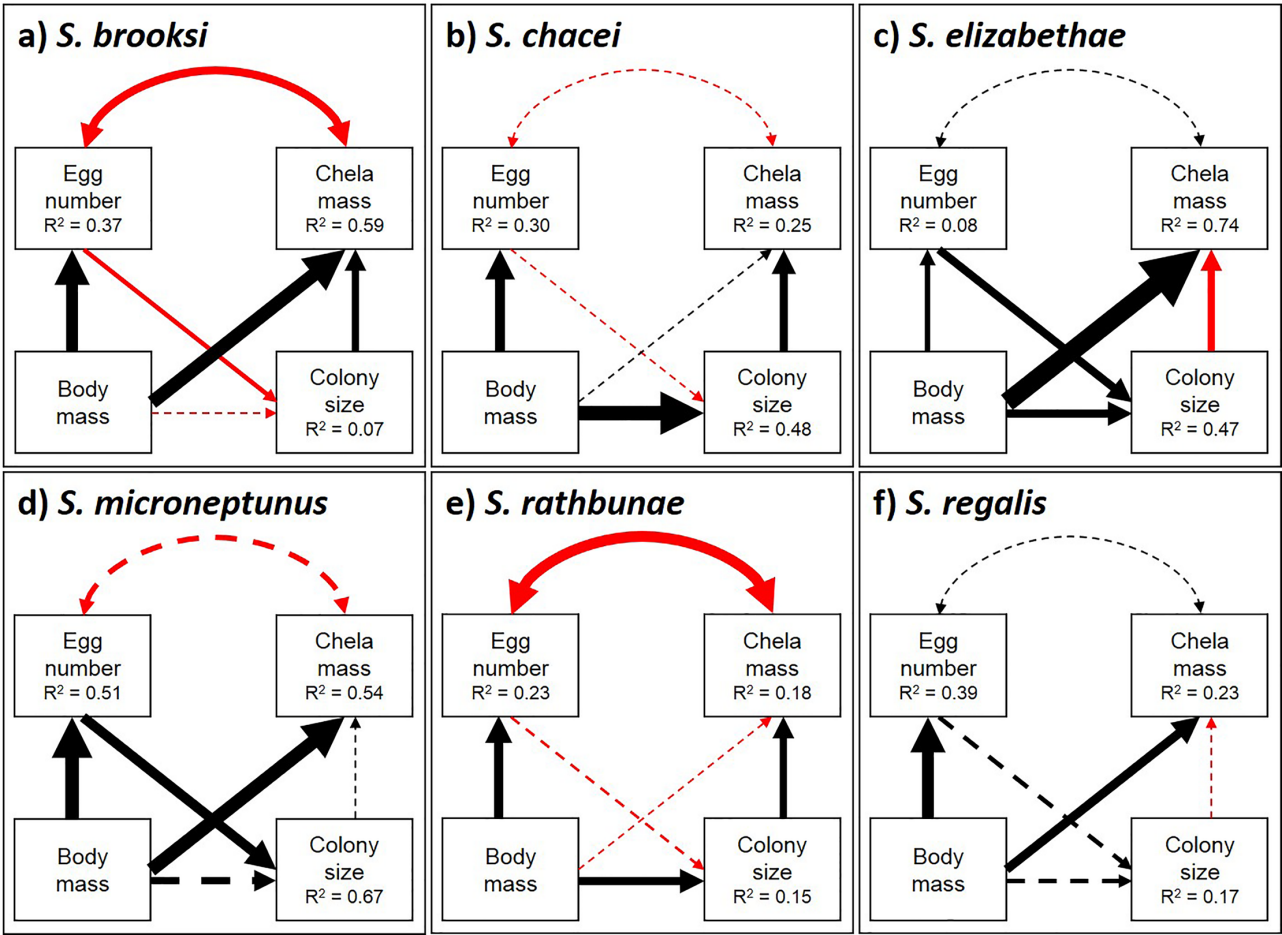
Results of structural equation models with variance explained (R^2^) for each pathway. The sign of the correlation is color coded (positive/negative, +/-, black/red) and arrow size is scaled by strength of the standardized path coefficients (S1 Table). Solid paths are significant (p ≤0.05) and dotted paths are non-significant.

The SEM results generally supported the hypothesized allometric relationships, with a few exceptions. The influences of body mass on major chela mass and of body mass on egg number were positive and significant in the majority of species, indicating regular allometric scaling of growth and reproduction (Fig 3; S2 Table). Major chela mass increased significantly with body mass in four of six species (*S*. *brooksi*, *S*. *elizabethae*, *S*. *microneptunus*, and *S*. *regalis*), and egg number increased significantly with body mass in all six species. The hypothesized trade-off between major chela mass and egg number was significant and negative in two of the six species analyzed: *S*. *brooksi* and *S*. *rathbunae*.

Additionally, colony size significantly increased with egg number in two species (*S*. *elizabethae* and *S*. *microneptunus*), but significantly decreased in *S*. *brooksi* (Fig 3). Colony size also significantly increased with body mass in three of six species (*S*. *chacei*, *S*. *elizabethae*, and *S*. *rathbunae*). The correlation between colony size and chela mass was positive and significant in three of the six analyzed species (*S*. *brooksi*, *S*. *chacei*, and *S*. *rathbunae*), but negative and significant in *S*. *elizabethae*.

**Fig 4:**
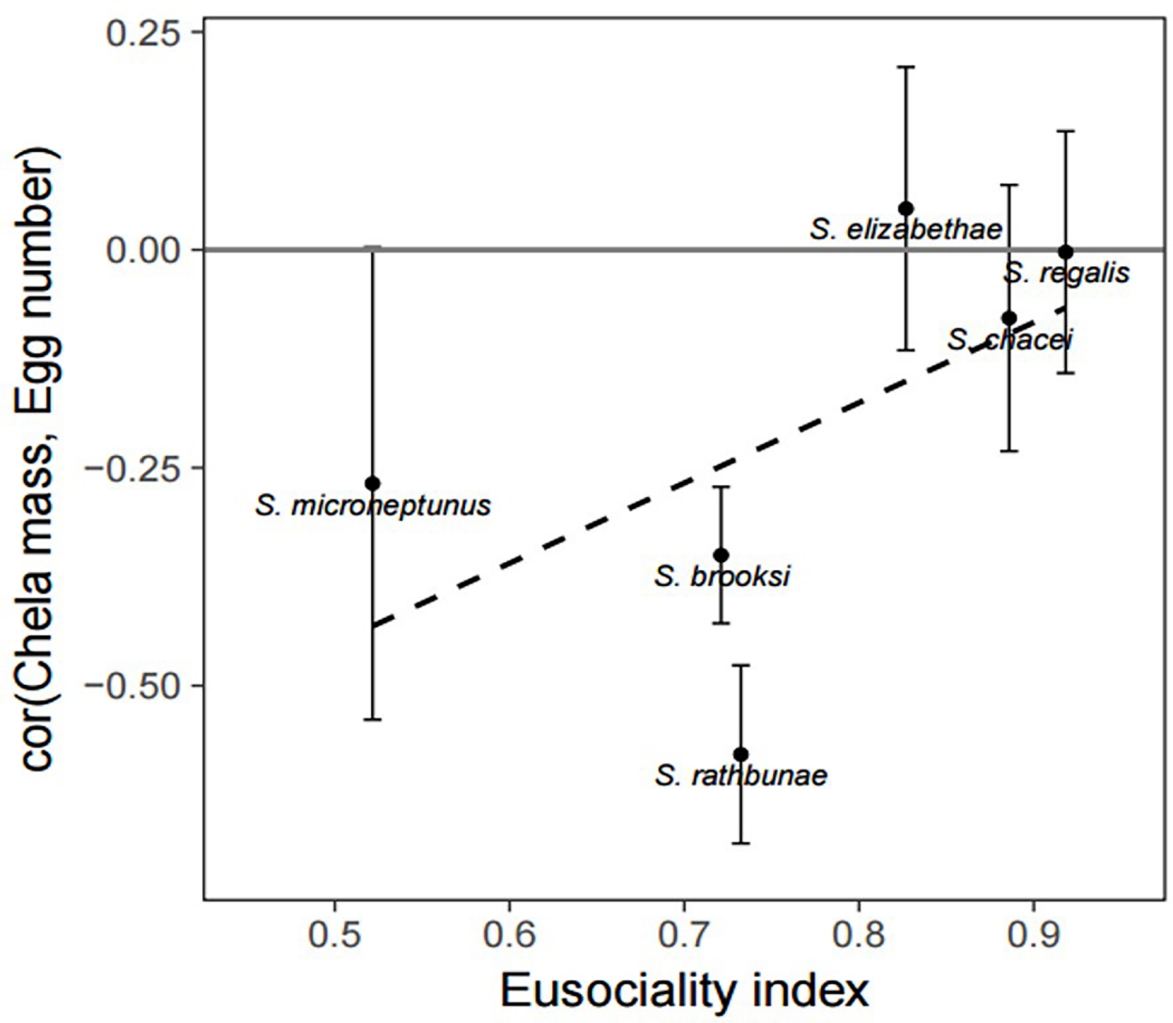
Relationship between eusociality index and bivariate correlation of chela mass and egg number. Recovered from the structural equation models representing the trade-off between reproduction (egg number) and defense (chela mass). Error bars represent +/- 1 standard error of the mean. The dashed line is the (non-significant) predicted fit from a simple linear regression.

Evaluation of the eusociality index identified three strongly eusocial species (*S*. *chacei*, *S*. *elizabethae*, and *S*. *regalis*) and three less eusocial species (*S*. *brooksi*, *S*. *microneptunus*, and *S*. *rathbunae*). The strength of the trade-off between chela mass and egg number trended negatively with Eusociality index, meaning that highly eusocial species showed a weaker dependence of fecundity on chela size, although this relationship was not statistically significant (*P* = 0.26, Fig 4). As predicted, the three species with the highest eusociality index demonstrated the weakest trade-off coefficients. The two species with the significantly negative trade-off, *S*. *brooksi* and *S*. *rathbunae*, were among the weakly eusocial species (Fig 4).

## Discussion

Of the three weakly-eusocial *Synalpheus* species, two showed evidence of the predicted trade-off between queen investment in reproductive output (egg number) and defensive weaponry (major chela mass), in the form of a significant negative correlation between the two (Fig 3A and 3E). The presence of a strong trade-off in the queens of *S*. *brooksi* and *S*. *rathbunae* supports our hypothesis that female-female competition for reproductive opportunity can drive investment in defense and reproduction. The third weakly eusocial species (*S*. *microneptunus*), showed the third strongest (albeit non-significant) negative correlation between egg number and major chela mass, behind *S*. *brooksi* and *S*. *rathbunae* (Fig 3D). Although our data rank *S*. *microneptunus* as the least eusocial, this is due to small colony size, not multiple queens. The strength of the trade-off is therefore likely a product of having small single-queen *S*. *microneptunus* colonies: with no rival reproducing females, *S*. *microneptunus* queens can invest heavily in reproduction to bolster small colonies.

In contrast, the strongly eusocial species (*S*. *chacei*, *S*. *elizabethae*, and *S*. *regalis*) demonstrated weakly negative or positive relationships between defense and reproduction (Fig 3B, 3C and 3F). The comparison among these two groups of species support our hypothesis that intra-sexual competition sets up a trade-off in queen allocation between reproduction and defense and that this trade-off is relaxed in highly eusocial species, in which a single female typically has reproductive monopoly. Notably, no species demonstrated a significantly positive relationship between chela mass and egg number, indicating that, in accordance with our initial hypotheses, both reproduction and defense cannot be simultaneously maximized.

Of the two species that exhibited the expected tradeoff, *S*. *brooksi* is a species whose colonies can have dozens of reproducing females. Although not directly included in this analysis, the number of reproducing females in each colony can mediate both an individual queen’s relatedness to other colony members and the likelihood of intra-colony competition that requires investment in defense. As mentioned above, however, multiple reproducing females within a colony may increase intra-sexual female competition within the colony [22], favoring a large major chela, and maintaining the trade-off. Regardless, the trade-off between reproduction and defense in *S*. *brooksi* and *S*. *rathbunae* suggests that eusocial queens must balance their energy investment according to social structure, kin aggregation, and ecological pressures.

The trend toward a weaker trade-off between chela mass and egg number in more highly eusocial species indicates that queens of such species, which tend also to be closely genetically related to other colony members [8], may have less need to maintain defensive weaponry, freeing resources for reproduction. Species with high reproductive skew can be considered the strongest manifestation of eusocial evolution, and thus more likely than other species to have already resolved and minimized the costs of a trade-off between reproduction and defense. While this trend was not statistically significant, probably on account of having a sample size of only *N* = 6 species, the simple linear regression with the eusociality index explained almost one-third of the variance in the trade-off *(R*^*2*^ = 0.31, Fig 4), suggesting that additional species may strengthen this relationship. The trade-off may also be dependent on the age of the sampled colonies, a variable that we were unable to characterize, since allometry changes somewhat with colony size within species [30]. Nonetheless, these results demonstrate strong potential for a link between the trade-off in reproduction and defense, and the development of eusociality.

Although many of the allometric relationships we documented supported our initial predictions, there was striking variation both within and among species. These differences suggest that queens of each species are affected by extrinsic factors not included in this study. For instance, given the nature of the sampling design, we could not include many ecological conditions, such as host-sponge volume, habitat availability, overall reef health, and even the biogeographic location in which the samples were collected, all of which may influence reproducing females of *Synalpheus* colonies. Additionally, our sampling protocols did not allow for longitudinal colony monitoring. Although it is challenging to simulate these marine microcosms *in-vitro*, future studies of *Synalpheus* ecology and behavior would benefit from lab-based, controlled longitudinal observations of colony formation and development. Furthermore, in this study, we assume that the only main source of colony recruits is queen reproduction and that emigration and immigration are negligible. However, given that host sponges have limited habitable space, *Synalpheus* colony growth likely diminishes or ceases entirely once they reach carrying capacity. At that point, we might expect to see colony reproduction via the dispersal of reproductively capable members or colony fission. Although colony reproduction is commonly observed in social insects, its prevalence in eusocial *Synalpheus* populations is unknown. The dispersal rate of adult *Synalpheus* seems low based on genetic homogeneity of colonies within sponges [8,41] but is nevertheless poorly understood, and may contribute to variation in colony size and success. The variation observed within and among these congeneric species suggests that different species adopt different strategies to develop and maintain social colony organization and that allometry of defense and reproduction are adjusted according to the social milieu.

## Conclusions

This study suggests that, in social shrimp, a queen’s investment in reproduction versus defense is mediated, in part, by social context, specifically the potential for intra-sexual conflict in the form of other reproducing females within a colony. We find evidence consistent with a trade-off in queen energy allocation between reproductive success and defense weaponry. The strength of this trade-off depends on colony size as well as reproductive skew, and varies across species. The queens of highly eusocial species tend to show a weaker trade-off, likely reflecting relaxed selection for maintaining defense capacity within large colonies of kin with more closely aligned reproductive interests. These findings suggest that the evolution of eusociality in shrimp has left a signal in the allometry of female reproduction and defense. As the sole eusocial marine genus, *Synalpheus* has much yet to teach us about the evolution of group-living and eusociality.

## Supporting information

S1 Data Raw data for specimen measurements and calculations. (CSV)

S1 File R script for reproducing analyses and figures. (R)

S1 Table Estimated path coefficients from structural equation models fit to each *Synalpheus* species. Operators denote: ~ = regression, ~~ = correlation. Estimated unstandardized (Unstd.) and standardized (Std.) path coefficients, and p-values for each correlation.
(XLSX)

S2 Table Mean values for calculated variables. (XLSX)
